# Study of Fine Needle Aspiration Cytology (FNAC) of Thyroid Gland According to the Bethesda System

**DOI:** 10.7759/cureus.37371

**Published:** 2023-04-10

**Authors:** Keval A Patel, Garima Anandani, Bhawana S Sharma, Riddhi A Parmar

**Affiliations:** 1 Pathology, GMERS Medical College, Vadnagar, IND; 2 Pathology, All India Institute of Medical Sciences, Rajkot, IND; 3 Pathology, Gujarat Adani Institute of Medical Sciences, Bhuj, IND

**Keywords:** histopathological correlation, cytopathology, thyroid, fine needle aspiration cytology, bethesda system

## Abstract

Background: Fine needle aspiration cytology (FNAC) of thyroid gland is a powerful diagnostic tool for thyroid nodules. The Bethesda System for Reporting Thyroid Cytopathology (TBSRTC) classifies thyroid FNAC findings into six categories. It is a standardized, simple, and convenient method of reporting which also provides guidelines for management.

Aims and Objectives: To study the cytomorphology of thyroid lesions and classify them as per TBSRTC. Determine the epidemiology and distribution of various thyroid lesions in our tertiary care hospital. Correlation of cytopathology with histopathological diagnosis in cases which were operated in our hospital.

Methods and Material: This is a prospective analytical study of 105 patients with clinically enlarged thyroid gland presenting at G.K. General Hospital, Bhuj during July 2018 to August 2020. FNAC smears of these patients were studied and correlated with histopathology wherever available.

Results: Out of a total 105 cases, 94 were non-neoplastic, eight were neoplastic, and three were unsatisfactory for evaluation. There were 94 cases in the benign category (category II), with colloid goiter being the most common cytological diagnosis (38 cases). There were no cases in categories III and V, respectively. On cytology, two cases in category IV were diagnosed as follicular neoplasm. Category VI had six cases comprising papillary carcinoma of thyroid (five cases) and medullary carcinoma of thyroid (one case). Out of a total 105 cases, 55 patients were operated in our center and hence their cytopathological findings were correlated with histopathological findings. Out of 55 operated cases, 45 cases (81.8%) had benign lesion and 10 cases (18.2%) were malignant. The sensitivity of FNAC was 70% and specificity was 100%.

Conclusions: Thyroid cytology proves to be a reliable, simple, and cost-effective first-line diagnostic procedure with high patient acceptance and with rare, usually easily treated and not life-threatening complications. The Bethesda system is very useful for a standardized and reproducible system of reporting thyroid FNAC. It satisfactorily correlates with the histopathological diagnosis and helps in comparing results amongst various institutes.

## Introduction

Thyroid disorders are widespread and can manifest as either a systemic condition like Grave's disease or a localized abnormality such as goiter or tumor mass. Thyroid nodules are a common clinical problem. It is important to differentiate benign from malignant nodules. Fine needle aspiration (FNA) is utilized as a safe, simple, and cost effective preoperative diagnostic technique for patients with thyroid nodules for triaging them into groups requiring invasive and non-invasive management [1]. FNA, which has excellent sensitivity, specificity, and diagnostic accuracy, is a frequently used method for diagnosing thyroid lesions [2-3].

“The Bethesda System for Reporting Thyroid Cytopathology” (TBSRTC) includes definitions, diagnostic/morphologic criteria, explanatory notes, and a brief management plan for each diagnostic category [4-5]. In this study we have analyzed fine needle aspiration cytology (FNAC) smears of thyroid gland according to 2017 revision of The Bethesda System for Reporting Thyroid Cytopathology and studied the correlation with histopathological diagnosis.

## Materials and methods

This is a prospective study that took place from July 2018 to August 2020 in the Department of Pathology at GK General Hospital, Gujarat Adani Institute of Medical Sciences, Bhuj. The ethical clearance for the study was approved by the Institutional Ethics Committee, Gujarat Adani Institute of Medical Sciences, Bhuj, with an IRB number of GAIMS/IEC/APPROVAL/25/2018. Informed consent was obtained from the patients.

*Inclusion criteria*: All patients with thyroid lesions, regardless of age or gender, who were admitted to wards or referred from the ENT and Surgery OPD to the Department of Pathology at GKGH Hospital Bhuj for cytological examination were included. Before obtaining a sample for cytological analysis, a comprehensive clinical examination was performed and detailed clinical history were taken from each patient.

The FNAs were performed using 22G or 23G needle, with or without the use of syringe for aspiration, utilizing imaging guidance wherever necessary. For Giemsa staining, some slides were air-dried, while others were immediately fixed in methanol for Hematoxylin and Eosin (H&E) and Papanicolaou (PAP) staining. FNAs were reported using TBSRTC, and the cancer risk according to the guidelines was communicated to the surgeon for further management.

Thyroidectomy specimens were fixed in 10% formalin for 12-18 h after gross morphological features were documented and the submitted tissue sections were processed for paraffin embedding. Thereafter 3-5-micron thick tissue sections were obtained and stained with H&E stain. Special stains such as PAS (Periodic Acid-Schiff) and Congo red were used as and when required. The cytological and histopathological findings were correlated. Microsoft Excel was used to enter all of the data. Tables, bar diagrams, and pie charts were used to interpret the results of the statistical analysis. Descriptive statistics like mean, median, standard deviation, range, sensitivity, specificity, positive predictive value (PPV) and negative predictive value (NPV) were calculated. PPV = TP/(TP + FP) × 100 and NPV = TN/(FN + TN) × 100 where TP is true positive, FP is false positive, TN is true negative, and FN is false negative.

## Results

**Figure 1:**
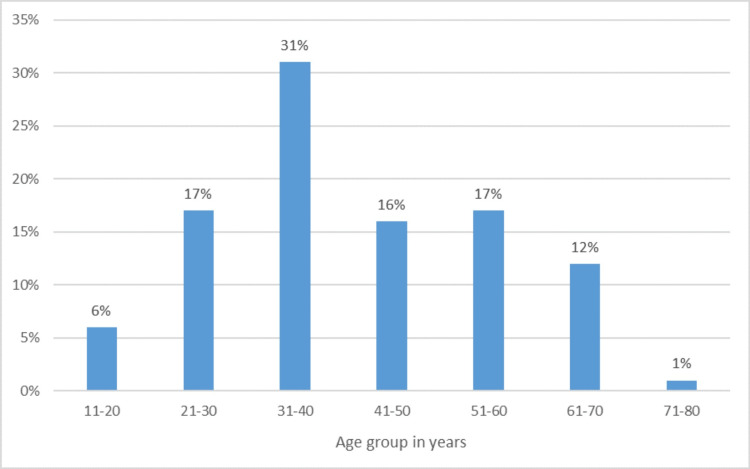
Distribution of patients according to age (N=105).

The FNAC smears of a total of 105 cases were examined. The age distribution ranged from 15 to 75 years. The patients' mean age was 43 years. The youngest patient was a 15-year-old female and the oldest was of 75 years. Figure 1 shows age distribution with percentage. Some 90 (86%) of the 105 patients with thyroid lesions were female, while 15 (14%) were male. The ratio of women to men was 6:1. 

Three aspirates (3%) out of 105 FNACs were inadequate for cytological analysis. As a result, they were labeled as "unsatisfactory smears" (Category I). These cases were advised a repeat FNA but they did not come for follow up or repeat FNA. The number of cases in each TBSRTC category is shown in Table 1.

**Table 1:** Categorization of cytopathological diagnosis with ROM and management recommendation according to Bethesda system (N=105). ROM, risk of malignancy; AUS/FLUS, atypia of undetermined significance or follicular lesion of undetermined significance; ND/UNS, nondiagnostic, unsatisfactory; FN/SFN, follicular neoplasm or suspicious for a follicular neoplasm; SFM, suspicious for malignancy

Categories	Number of cases	Percentage (%)	% ROM	Management recommendation
Category I ND/UNS	03	2.8	5–10	Repeat FNA with ultrasound guidance
Category II benign (B)	94	89.6	0–3	Clinical and ultrasonographic follow-up
Category III (AUS/FLUS)	00	00	6–18	Repeat FNA, molecular testing or lobectomy
Category IV (FN/SFN)	02	1.9	10–40	Molecular testing, lobectomy
Category V SFM	00	00	45–60	Near-total thyroidectomy or lobectomy
Category VI Malignant (M)	06	5.7	94–96	Near-total or total thyroidectomy
Total	105	100		

**Figure 2:**
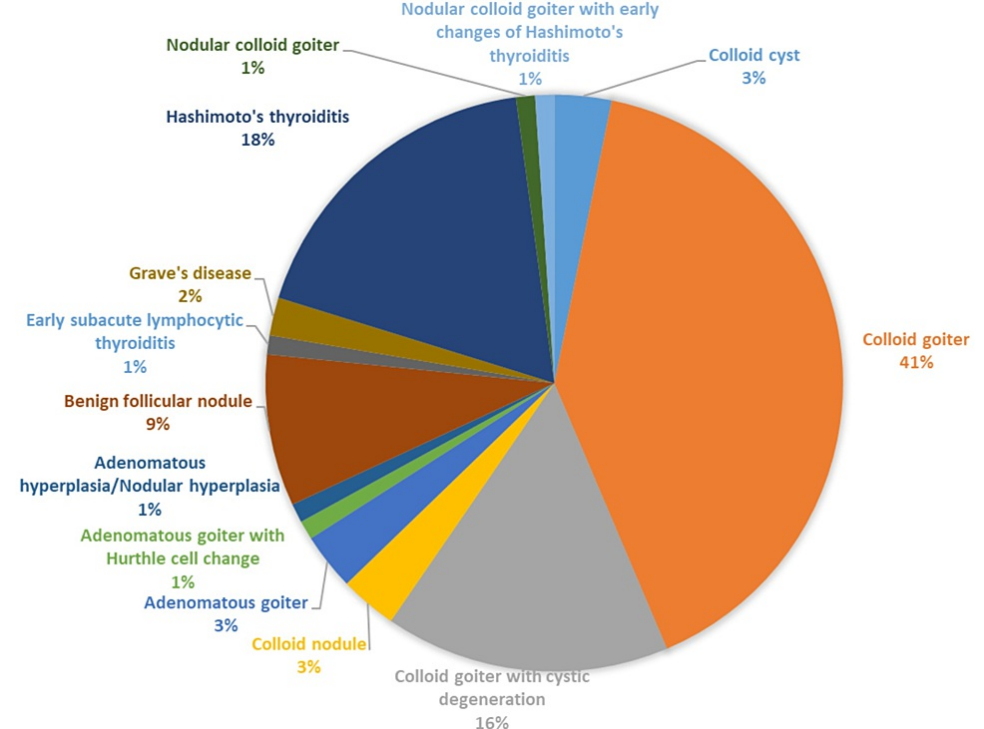
Distribution of patients according to Category II (N=94).

Figure 2 shows details of Benign (TBSRTC category II) lesions. The maximum numbers of cases (94) were found in category II.

**Figure 3:**
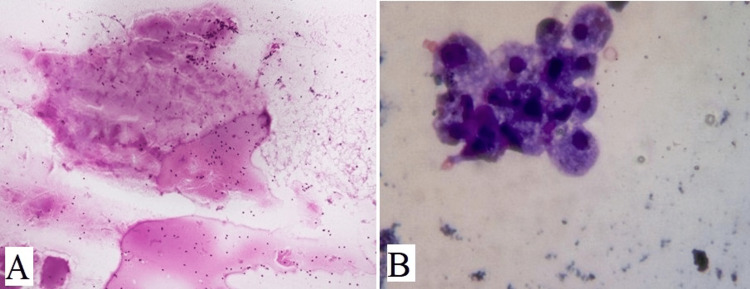
(A) shows thin, watery pink color colloid with variable density, cracks, and folds (H&E, 100x), (B) shows cyst macrophages in colloid goiter with cystic degeneration (MGG, 1000x).

**Figure 4:**
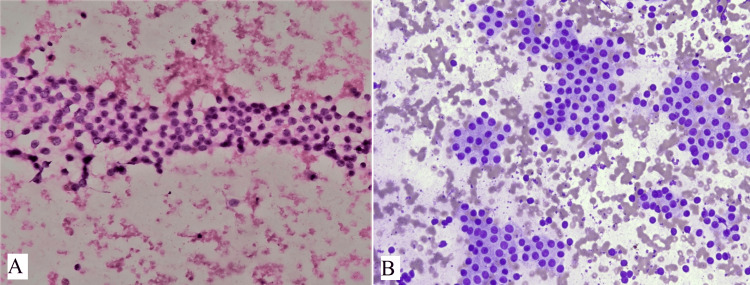
(A) shows benign follicular cells having round nuclei and scant to moderate amount of cytoplasm (H&E, 400x), (B) shows benign follicular nodule having monolayered sheets of evenly spaced follicular cells arranged in honeycomb-like pattern (MGG, 400x).

**Figure 5:**
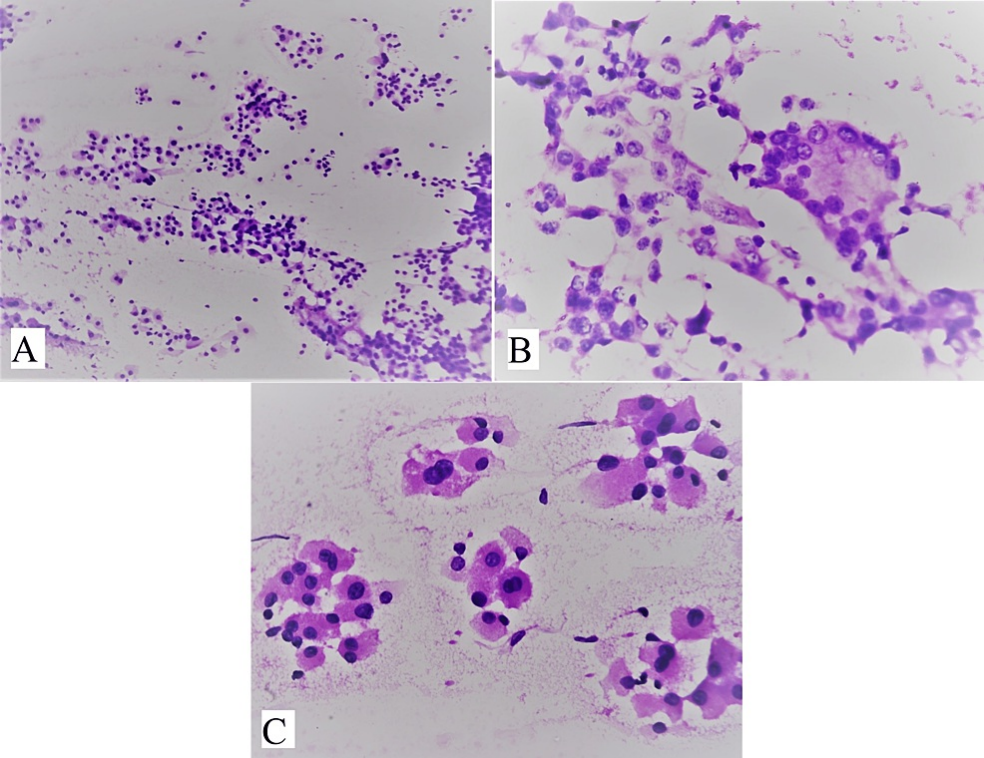
(A) shows mixed population of Hurthle cells (oncocytes) and lymphocytes (H&E, 100x), (B) shows multinucleated giant cell in a case of Hashimoto’s thyroiditis (H&E, 400x), (C) shows Hurthle cells having abundant granular cytoplasm, large nuclei, and prominent nucleoli with mild anisonucleosis (H&E, 400x).

Out of 94 cases, 38 cases were colloid goiter showing thin, and watery pink color colloid with variable density with cracks and folds (Figure 3A). Cases with fluid aspirate from cysts showed cyst macrophages and hemosiderin-laden macrophages were reported as colloid goiter with cystic degeneration (Figure 3B). Cases of benign follicular nodules had moderate cellularity, consisting of benign follicular epithelial cells having round to oval nuclei with uniform granular chromatin and scant to moderate amount of cytoplasm (Figure 4A). Follicular cells were arranged in monolayered sheets and showed honeycomb-like appearance (Figure 4B). Some 17 cases of Hashimoto’s thyroiditis showed moderate cellularity comprising follicular cells arranged in microfollicles. There was a mixed population of Hurthle cells and lymphocytes along with occasional plasma cells in the background (Figure 5A). Multinucleated giant cells were also seen (Figure 5B). Hurthle cells have distinct cell border, abundant finely granular cytoplasm, and large nuclei. Some Hurthle cells showed prominent nucleoli with mild anisonucleosis (Figure 5C).

**Figure 6:**
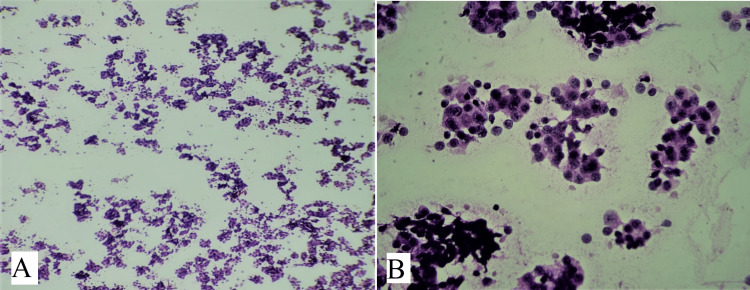
(A) shows moderate cellularity comprising uniform follicular cells arranged in crowded clusters of microfollicles (H&E, 100x), (B) shows follicular cells in crowded, microfollicular, or rosette arrangements with mild size variation, vesicular chromatin, and prominent nucleoli (H&E, 400x).

In Category III (AUS/FLUS), no cases were identified. In Category IV (FN/SFN), two cases were diagnosed as follicular neoplasm (Table 2). Microscopic features showed moderate to marked cellularity comprised of uniform follicular cells arranged in crowded clusters, microfollicles and singly scattered. Background showed scant colloid material (Figure 6A). The follicular cells showed crowding or rosette-like arrangement having normal size or mildly enlarged, with scant or moderate amount of cytoplasm. Nuclei were round with hyperchromasia, occasional prominent nucleoli, and vesicular chromatin (Figure 6B). None of the patients had a Category V diagnosis (suspicious for malignancy).

**Table 2:** Distribution of patients according to neoplastic lesions (N=08).

Type of category	Number	Percentage
Atypia of undetermined significance (Category III)	00	00
Follicular neoplasm/suspicious for a follicular neoplasm (FN/SFN) (Category IV)	02	25
Suspicious for malignancy (Category V)	00	00
Category VI		
a) Papillary carcinoma	05	62.5
b) Medullary carcinoma	01	12.5
Total	08	100

**Figure 7:**
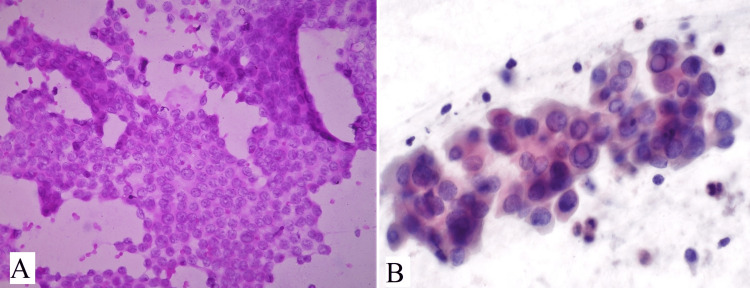
(A) shows sheets of cells with large, crowded nuclei, powdery chromatin with many longitudinal nuclear grooves and prominent nucleoli (H&E, 400x), (B) shows pseudo nuclear inclusion in a case of papillary carcinoma of thyroid (PAP stain, 1000x).

**Figure 8:**
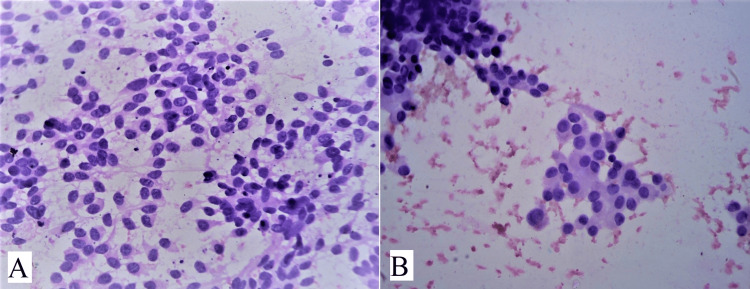
(A) shows cluster of spindle cells and few plasmacytoid cells with mild nuclear pleomorphism and visible nucleoli in case of medullary carcinoma of thyroid (H&E, 400x), (B) shows plasmacytoid cells in cluster in a case of medullary carcinoma of thyroid (H&E, 400x).

In Category VI (malignant), five cases were diagnosed as papillary carcinoma and one case as medullary carcinoma of thyroid. On microscopy, papillary carcinoma showed neoplastic cells having high N/C ratio, crowded oval nuclei, prominent nucleoli, and powdery chromatin with longitudinal nuclear grooves (Figure 7A). Intranuclear cytoplasmic pseudoinclusions are also seen (Figure 7B). Medullary carcinoma of thyroid showed moderate to marked cellularity comprising spindle cells and medium-sized polygonal cells. There was a cluster of spindle cells having cytoplasmic processes with oval nuclei, smooth nuclear membranes, granular chromatin, and prominent nucleoli (Figure 8A). Plasmacytoid or polygonal cells were also seen with granular chromatin and small visible nucleoli (Figure 8B). Congo red stain was also used to identify presence of amyloid stroma and differentiate from colloid.

Out of a total 105 cases, 55 patients underwent surgery for which histopathological findings were available. Among 55 cases, 45 cases (81.8%) had benign lesions and 10 cases (18.2%) had malignant lesions. Goiter was the most prevalent benign lesion in the present study, comprising colloid goiter (20 cases), colloid goiter with cystic degeneration (08 cases), multinodular goiter (05 cases) and adenomatous goiter (02 cases), followed by Hashimoto’s thyroiditis (08 cases), Grave’s disease (01 case), and follicular carcinoma (01 case). Out of 10 malignant lesions, there were five cases of papillary thyroid carcinoma (50%), two cases of follicular variant of papillary thyroid carcinoma (FVPTC) (20%), one case of noninvasive follicular thyroid neoplasm with papillary-like nuclear features (10%), one case of medullary carcinoma (10%), and one case of follicular carcinoma (10%).

The cytohistological correlation of thyroid lesions is shown in Table 3. Out of 47 histologically proven benign cases, 44 were benign on cytology as well. One case of follicular neoplasm was diagnosed as follicular adenoma on histology, and the other case was diagnosed as follicular carcinoma on histology. Although the diagnosis of follicular carcinoma needs histopathological evidence of capsular or vascular invasion by the tumor cells, this was considered as a positive correlation. Six cases fell into the malignant category, and histopathological confirmation of their malignancy gave them 100% specificity. Out of 10 histologically proven malignant cases, seven were also malignant on cytology. Three of these cases were found to be benign on cytology but malignant on histopathology. Histopathology revealed a FVPTC in a case of nodular goiter that had been diagnosed on cytology. On cytology, the second case was found to be a benign follicular nodule; however, histopathology revealed it to be a noninvasive follicular thyroid neoplasm with papillary-like nuclear features and the third one was reported as adenomatoid hyperplasia/nodular hyperplasia on cytology but proven to be FVPTC on histopathology.

**Table 3:** Comparison of cytological diagnosis with final histopathology with remarks and incidence of malignancy in each Bethesda Category (n=55). AUS/FLUS, atypia of undetermined significance/follicular lesion of undetermined significance; FN/SFN, follicular neoplasm/suspicious for follicular neoplasm

Bethesda category	Number of cytology cases	Number of cases with surgical follow-up on biopsy	Histopathological diagnosis	Number	Remark	Number of cases positive for malignancy, n (%)
1. Nondiagnostic	03	00	-	-	-	-
2. Benign	94	47	Colloid goiter	20	TN	03 (6.4)
			Colloid goiter with cystic degeneration	08	TN	
			Multinodular goiter	05	TN	
			Adenomatous goiter	02	TN	
			Hashimoto’s thyroiditis	08	TN	
			Grave’s disease	01	TN	
			NIFTP	01	FN	
			FVPTC	02	FN	
3. AUS/FLUS	00	00	-	-	-	-
4. FN/SFN	02	02	Follicular adenoma	01	TN	01 (50)
			Follicular carcinoma	01	TP	
5. Suspicious for malignancy	00	00	-	-	-	-
6. Malignant	06	06	Papillary carcinoma	05	TP	06 (100)
			Medullary carcinoma	01	TP	
Total	105	55	-	55		

## Discussion

The FNAC has been used worldwide for over four decades as a powerful diagnostic tool. The use of FNAC is justified owing to the procedure being inexpensive, minimally invasive, with minimal complications, and for giving an early preoperative diagnosis for most of the thyroid lesions. The keys to a successful diagnosis that paves the way for efficient surgical treatment of thyroid masses are the location of the target lesion, a thorough search for malignant cells, and repeat FNAC. It is essential to distinguish between benign and malignant thyroid nodules because malignancy requires surgery, whereas a benign thyroid mass usually requires strict patient follow-up unless rarely causing airway obstruction and difficulty in breathing or eating requiring removal.

Table 4 shows comparative study of our study's age distribution with other studies. In our study, the age of patients ranged from 15 to 75 years with mean age of 43 years. Similar mean age was seen in other studies by Naz et al. [6] (39.7), Gupta et al. [7] (38.7), Bhat et al. [8] (36), Dhamecha et al. [9] (38.53), and Khatib et al. [10] (40.7). The mean age in a study of Park et al. [11] is 50 years, which is higher as compared with our study and other reported series.

**Table 4:** Comparison of age-distribution of cases amongst various studies.

Authors and References	Age range (years)	Mean age (years)
Naz et al. [6]	14-84	39.7
Gupta et al.[7]	22-58	38.7
Bhat et al. [8]	11-73	36
Dhamecha et al. [9]	7-75	38.53
Khatib et al. [10]	6-85	40.7
Park et al. [11]	14-86	50
Present study	15-75	43

Thyroid lesions are more prevalent in females; in our study, the female to male ratio was 6:1. Similar ratio was seen in studies by Melo-Uribe et al. [12] (7.9:1) and Handa et al. [13] (5.3:1). The female to male ratio in the study of the Naz et al. [6], Bhat et al. [8], and Park et al. [11] is less as compared to our study (Table 5).

**Table 5:** Comparison of gender-distribution of cases in different studies.

Name of the study	Females	Males	Total cases	Female: Male ratio
Naz et al. [6]	413	115	528	3.6:1
Bhat et al. [8]	400	200	600	2:1
Park et al. [11]	1217	321	1538	3.8:1
Melo Uribe et al. [12]	174	22	196	7.9:1
Handa et al. [13]	366	68	434	5.3:1
Present study	90	15	105	6:1

In our study the adequacy rate was 97%. The adequacy rate of the present study was comparable with studies by Naz et al. [6] (94%), Bhat et al. [8] (93.3%), Khatib et al. [10] (99%), Melo-Uribe et al. [12] (95.6%), and Handa et al. [13] (94.9%).

In our study maximum cases were non neoplastic on cytological diagnosis. Non-neoplastic to neoplastic ratio was 11.7:1 in our study; this was in accordance with studies by Dhamecha et al. [9] (10.2:1) and Handa et al. [13] (12.3:1). The ratio in studies of Bhat et al. [8] and Khatib et al. [10] is 7.2:1 and 7.6:1 respectively, which is lower as compared with the present study (Table 6).

**Table 6:** Comparison of non-neoplastic and neoplastic lesions.

Name of the study	Non-neoplastic	Neoplastic	Ratio
Bhat et al. [8]	492	68	7.2:1
Dhamecha et al. [9]	328	32	10.2:1
Khatib et al. [10]	252	33	7.6:1
Handa et al. [13]	381	31	12.3:1
Present study	94	08	11.7:1

In the present study, the percentage of cases in each category of TBSRTC was in accordance with the studies of Khatib et al. [10] and Mondal et al. [14]. In contrast to Park et al. [11] and Jo et al. [15] studies, the prevalence of category I lesions was significantly lower as a result of repeating the FNA if the results were inconclusive. The current study did not find any lesions in categories III or V (Table 7).

**Table 7:** Comparison of incidence of Bethesda categories. Cat, Category

Name of the study	Bethesda Cat I (%)	Bethesda Cat II (%)	Bethesda Cat III (%)	Bethesda Cat IV (%)	Bethesda Cat V (%)	Bethesda Cat VI (%)
Bhat et al. [8]	6.6	82	2	2.5	1.6	5.1
Dhamecha et al. [9]	10	82	1.25	5.75	0.25	0.75
Khatib et al. [10]	0.68	88	3.4	4.5	1.4	2.06
Park et al. [11]	13.3	40.6	9.1	0.4	19.3	17.3
Mondol et al. [14]	1.2	87.5	1	4.2	1.4	4.7
Jo et al. [15]	18.6	59	3.4	9.7	2.3	7
Present study	03	89	0	02	00	06

Out of the 105 total thyroid FNACs, only 55 underwent surgery and were correlated with FANC findings. Table 3 shows the cyto-histopathological correlation of thyroid swelling and the incidence of malignancy in various TBSRTC categories. Out of 47 benign cases, three (6.4%) cases were diagnosed as malignant on histopathology, with two cases diagnosed as FVPTC and one as a noninvasive follicular thyroid neoplasm with papillary like nuclear features (NIFTP). The frequency of malignancies in each category of our study is shown in Table 8 as a percentage. Except for group IV, there was concordance across all categories [14-17]. The current study had only two cases in category IV, one of which was diagnosed as a follicular adenoma and the other as a follicular carcinoma.

**Table 8:** Comparison of malignancy rates. Cat, Category

Study	Bethesda Cat I (%)	Bethesda Cat II (%)	Bethesda Cat III (%)	Bethesda Cat IV (%)	Bethesda Cat V (%)	Bethesda Cat VI (%)
Mondol et al. [14]	0	4.5	20	30.6	75	97.8
Jo et al. [15]	8.9	1.1	17	25.4	70	98.1
Yassa et al. [16]	10	0.3	24	28	60	97
Yang et al. [17]	10.9	7.3	13.5	32.2	64.7	98.6
Present study	-	6.4	-	50	-	100

The FNAC is a highly sensitive and specific method of evaluating thyroid nodules for malignancy. The sensitivity, specificity, PPV, and NPV in this study are 70%, 100%, 100%, and 93.8%, respectively (Table 9). In the present study, specificity, PPV, and NPV were all in concordance with studies by Gupta et al. [7], Handa et al. [13], and Kadam et al. [18]. In a study by Tabaqchali et al. [19], the specificity and PPV are 67% and 65.5%, respectively, which are lower than in our study. The fact that both the specificity and PPV were 100% indicates that our institution's FNA was effective. The small sample size and the small size of a malignant lesion may have contributed to the low sensitivity rate.

**Table 9:** Comparison of statistical indices. PPV, positive predictive value; NPV, negative predictive value

Study	Sensitivity	Specificity (%)	PPV (%)	NPV (%)
Gupta et al. [7]	80	86.6	80	86.6
Handa et al. [13]	97	100	96	100
Kadam et al. [18]	20	100	100	91.1
Tabaqchali et al. [19]	86.8	67	65.5	87.5
Present study	70	100	100	93.8

Limitations

The number of patients we could include in this study was limited due to time constraints. Many patients with thyroid nodules who were reported as benign on cytology, did not undergo surgery. Hence, assessing the final histopathological diagnosis in such cases was not possible. A reliable false-negative rate can only be determined if all patients, regardless of FNA results, undergo surgery; this was not feasible. A cell block obtained through aspiration in malignant or suspicious for malignant cases, may be more useful for ancillary techniques such as immunocytochemistry, which leads to a more accurate diagnosis and subsequent management. But this was not performed in our study.

## Conclusions

The FNAC is an extremely useful, competent, and cost-effective method of investigation for thyroid lesions having a high sensitivity, specificity, and accuracy especially in developing country like India having less resources. Hence it can be used as a first line of pathological investigation which can help in identifying patients who will require non-invasive or invasive management. The Bethesda system is very useful six-tiered standardized system of reporting thyroid cytopathology by decreasing the diagnostic discrepancies and facilitating diagnostic correlation with the histopathological excisions. This helps in uniformity and better understanding between the cytopathologist and the treating physician leading to a rational development of management plans.
